# Synthesis of Macrocyclic Bis-Hydrazone and Their Use in Metal Cations Extraction

**DOI:** 10.5402/2012/208284

**Published:** 2012-02-08

**Authors:** Farouk Kandil, Mohamad Khaled Chebani, Wail Al Zoubi

**Affiliations:** Department of Chemistry, Faculty of Science, University of Damascus, Damascus, Syria

## Abstract

Two new macrocyclic hydrazone Schiff bases were synthesized by reaction of succindihydrazide and adipdihydrazide with acetylacetone. Hydrazones have been characterized by elemental analyses and IR, mass, ^1^H NMR, and ^13^C NMR spectral data. Hydrazones have been studied by liquid-liquid extraction towards the s-metal ions (Li^+^, Na^+^, and K^+^) and d-metal ions (Cu^2+^ and Cr^3+^) from aqueous phase to organic phase. The effect of chloroform and dichloromethane as organic solvents over the metal chlorides extraction was investigated at 25 ± 0.1°C by using flame atomic absorption. We found differences between the two solvents in extraction selectivity.

## 1. Introduction

Hydrazones are special class of compounds in the Schiff bases family. They are characterized by the presence of the following

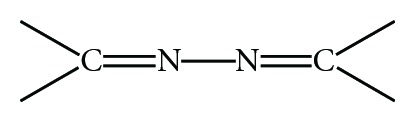
(1)
Hydrazone Schiff bases of acyl, aroyl, and heteroaroyl compounds have an addition donor sites like C=O. The additional donor sites make them more flexible and versatile. This versatility has made hydrazones good polydentate chelating agents that can form a variety of complexes with various transition and inner transition metals and have attracted the attention of many researchers [1]. 

A wide varieties of hydrazones and their metal complexes have been studied because of their important properties which can be used in different applications, such as, extraction of some metal ions [2], microdetermination of metal ions [2], determination of titanium in bauxite, Portland cement, amphibolites granites [3], and different biological activities, such as antimicrobial [2, 4–7], antifungal [8, 9], antitumor [10, 11], and insecticides [12]. For these applications, we are extending this field of compounds for synthesising novel macrocyclic hydrazones. 

Five dissymmetric tridentate Schiff base ligands containing a mixed donor set of ONN and ONO were prepared by the reaction of benzohydrazide with the appropriate salicylaldehyde and pyridine-2-carbaldehyde and characterized by FT-IR, ^1^H NMR, and ^13^C NMR [13]. 

Inoue et al. [14] reported the synthesis and spectroscopic characterization of complexes of Ni^2+^, Cu^2+^, Zn^2+^, and Cd^2+^ with hydrazones Schiff bases derived from 6-amino-5-formyl-1,3-dimethyluracil, nicotinic and isonicotinic acids hydrazides. Avaji et al. [15] reported the synthesis and characterization of a macrocyclic hydrazone Schiff bases prepared by condensation of 1,4-dicarbonyl phenyl dihydrazide with 2,6-diformyl-4-methyl phenol. Emara et al. reported [16–18] formation of binuclear complexes for the ligands derived from 4,6-diacetylresorcinol, where the ligands were prepared by condensation of 4,6-diacetylresorcinol (DAR) with oxalyldihydrazine (ODH) in the molar ratios (1 : 1) and (1 : 2) to afford the corresponding hydrazone, H_6_La and H_4_Lb, ligands, respectively. Otomo and Nakayama et al. [19] reported formation of three terdentate hydrazones, all containing the 1-phthalazino grouping in the hydrazine moiety but differing in the heterocyclic substituent in the aldehyde moiety, where the ligands were used as analytical reagents for palladium(II), the optimal conditions for the extractive spectrophotometric determination of palladium(II) in the presence of chloride ions being deduced. These compounds were highly selective and sensitive reagents for palladium(II), since they were not extracted into chloroform from sulfuric acid solutions and did not react with other platinum group metals. Odashima and Ishii [20] reported the synthesis and spectroscopic characterization of four new hydrazones, 2-pyridinecarbaldehyde 3-nitro-2-pyridylhydrazone, 2-pyridinecarbaldehyde 3,5-dinitro-2-pyridylhydrazone (PA-3,5-NPH), 2-quinolinecarbaldehyde 5-nitro-2-pyridylhydrazone, and 6-phenanthridinecarbaldehyde 5-nitro-2-pyridylhydrazone. Also useful information on the molecular design of the hydrazone reagent was obtained. A highly sensitive and practical extraction-spectrophotometric method for the determination of nickel with PA-3,5-NPH developed and applied successfully to the determination of nickel in steel samples. 

**Scheme 1:**
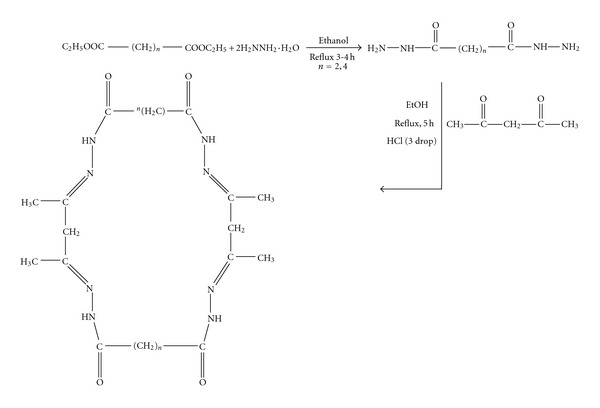
Synthesis of macrocyclic hydrazone Schiff bases.

In the context of the above applications, we have reported here the synthesis and characterization of novel macrocyclic hydrazone Schiff bases and their extraction study of some transition metal cations. The structure of Schiff bases and scheme synthesis is shown in Scheme 1.

## 2. Experimental

### 2.1. Reagents and Apparatus

All the chemicals used were of AnalaR grade and procured from Sigma-Aldrich and Fluka. Metal salts were purchased from E. Merck and were used as received. Distilled water was used in extraction experimentals. The solvents were saturated with each other before use in order to prevent volume changes of the phases during extraction.

The C, H, and N were analyzed on a Carlo-Erba 1106 elemental analyzer. The IR spectra was recorded on Jusco 300 instrument in KBr pellets. ^1^H and ^13^C-NMR spectra of ligands in CDCl_3_ solution were recorded on a Bruker DT-400 MHz spectrometer, and chemical shifts are indicated in ppm relative to tetramethylsilane. Mass spectra were recorded using a KRATOS MS50TC spectrometer.

AA 929 Unicam Spectrometer was used for FAAS measurements with an air-acetylene flame. A pH meter (Metrohm 691 pH Meter) was also used. All extractions experiments were performed by using a mechanical flask agitator in 50 cm^3^ stoppered glass flasks.

### 2.2. Synthesis of Diethyl Succinate

A succinic acid (9.19 mmol) in absolute dry ethanol (60 mL) containing 2-3 drops of concentrated H_2_SO_4_ was refluxed, continuing the heating for 20–30 minutes. When the reflux time is complete, remove the flask from the heating mantle, allow it to cool briefly. Then, the reaction mixture was poured onto ice cold water. Place about 5 mL chipped ice in a 50-mL beaker and mix it with 15 mL 10% aqueous Na_2_CO_3_ (sodium carbonate) (the pH should be above 7. If it is not, add more (10%) Na_2_CO_3_ dropwise until the solution is basic). The ester thus obtained was extracted by diethyl ether. Then dry the ether solution in the centrifuge tube by adding several spatula-tip loads of anhydrous Na_2_SO_4_ [17]. 

### 2.3. Synthesis of Diethyl Adipate

Adipic acid (7.92 mmol) in absolute dry ethanol (60 mL) containing 2-3 drops of concentrated H_2_SO_4_ was refluxed, then continue the heating for 20–30 minutes. When the reflux time is complete, remove the flask from the heating mantle, allow it to cool briefly. Then, the reaction mixture was poured onto ice cold water. Place about 5 mL chipped ice in a 50-mL beaker and mix it with 15 mL 10% aqueous Na_2_CO_3_ (sodium carbonate)(the pH should be above 7. If it is not, add more 10% Na_2_CO_3_ dropwise until the solution is basic). The ester thus obtained was extracted by diethyl ether. Then dry the ether solution in the centrifuge tube by adding several spatula-tip loads of anhydrous Na_2_SO_4_ [17].

### 2.4. Synthesis of Succinic Acid Dihydrazide

Mixture of diethyl ester of succinic acid (2.22 g) and hydrazine hydrate (98% 2 cc) in ethanol (80 mL) was refluxed for 4-5 h. The reaction mixture was allowed to cool in room temperature, then the cooled solution was poured onto ice cold water. The dihydrazide of Succinic acid thus obtained was filtered and recrystallized from ethanol.

### 2.5. Synthesis of Dihydrazide of Adipic Acid Dihydrazide

A mixture of diethyl ester of adipic acid (2.22 g) and hydrazine hydrate (98% 2 cc) in ethanol (80 mL) was refluxed for 4-5 h. The reaction mixture was allowed to cool in room temperature, then the cooled solution was poured onto ice cold water. The dihydrazide of adipic acid obtained was filtered and recrystallized from ethanol.

### 2.6. Synthesis of Macrocyclic Succinic Acid Hydrazone Ligand(I)

The hot ethanolic solution (20 mL), of acetylacetone (2 mmol, 0.2 g), and a hot ethanolic solution (20 mL), of dihydrazide of succinic acid (2 mmol, 0.292 g), were mixed slowly with constant stirring. This mixture was refluxed at ~75°C for 9 h in presence of few drops of concentrated hydrochloric acid (pH~3). The reaction mixture was allowed to cool in room temperature, then the cooled solution was poured onto ice cold water. On cooling, white-colored precipitate is separated out, filtered, washed with cold EtOH, and dried under vacuum over P_2_O_5_ yield 50%. Elemental analysis found % (found atomic mass 420 amu), C 51.14 H 5.64; N 26.53 for C_18_H_28_N_8_O_4_.

### 2.7. Synthesis of Macrocyclic Adipic Acid Hydrazone Ligand(II)

The hot ethanolic solution (20 mL), of acetylacetone (2 mmol, 0.2 g), and a hot ethanolic solution(20 mL), of dihydrazide of adipic acid (2 mmol, 0.348 g), were mixed slowly with constant stirring. This mixture was refluxed at ~75°C for 9 h in presence of concentrated hydrochloric acid (pH~3). The reaction mixture was allowed to cool in room temperature, then the cooled solution was poured onto ice cold water. On cooling, white-colored precipitate is separated out, filtered, washed with cold EtOH, and dried under vacuum. Yield 55%. Elemental analysis found % (found atomic mass 420 amu), C 55.69 H 6.66; N 22.62 for C_22_H_36_N_8_O_4_.

### 2.8. Extraction Procedure

Aqueous solutions containing 1.5 × 10^−3^ mol L^−1^ metal chloride in appropriate buffer were equilibrated with equal volumes of the chloroform and dichloromethane solutions of the ligand 4 × 10^−4^ M by shaking in a mechanical shaker at 25°C. Optimum equilibration time was determined for this system. In most cases, distribution equilibrium was attained in less than 180 min and a shaking time of 120 min. The ionic strength of the aqueous phase was 0.1 M KCl in all experiments except those in which the effect of ionic strength was studied. After agitation, the solutions were allowed to stand for 120 min. The copper(II) and chrome(III) concentrations of the aqueous phase were determined by FAAS and that of the organic phase from the difference by considering the mass balance. The pH of aqueous phase was recorded as equilibrium pH.

## 3. Results and Discussion

The preparation of two ligands containing nitrogen and oxygen donor atoms are shown in Scheme 1. The structures of new compounds were characterized by a combination of IR, MS, ^1^H NMR, and ^13^C NMR spectral data. Compounds were synthesized according to reported procedures [17].

**Ligand(I)**

The spectrum showed a strong bands at 1715 and 1587 cm^−1^ in the spectrum of the Schiff base assigned to *υ*(C=O) of carbonyl and azomethine *υ*(C=N) vibrations, respectively. An intense band at 3111 cm^−1^ is due to the –NH-vibrations of the hydrazine group, a broad medium intense band was at 2930 cm^−1^ due to methylene groups, and the band at 1068 cm^−1^ is assigned to hydrazinic *υ*(N–N) of the ligand [21–25].

**Figure 1:**
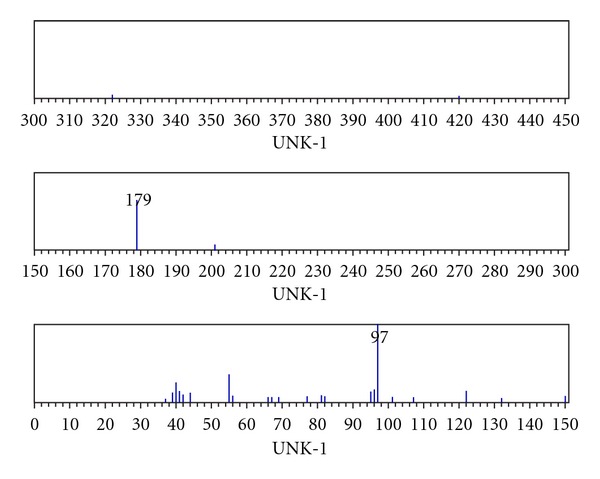
Mass spectra of Ligand(I).

In the electron impact spectrum (Figure 1) of the ligand, we confirm the probable formula by showing a peak at 420 amu, corresponding to macrocyclic moiety [(C_18_H_28_N_8_O_4_), calculated atomic mass 420]. The series of peaks in the range, that is, 37, 40, 41, 42, 44, 55, 95, 96, 97, 101, 121, 132, 150, 179, 201 amu, and so forth, may be assigned to various fragments. Their intensity gives an idea of stability of fragments (Figure 1).

In the ^1^H NMR spectrum the ligand(I) exhibits signals at 2.24 ppm due to CH_3_–C(6H), 2.54 due to –CH_2_(4H), 3.55 due to –CH_2_–CO–(8H) and at 5.9 ppm due to NH protons (Figure 3(a)).

In the ^13^C NMR spectrum (Figure 3(b)) of ligand(I), and indicated new resonances are 13.80, 14.58(–CH_3_), 23.74, 34.90(CH_2_), 110.96, 143.99(CH_2_CO), 151.77(C=N), 173.71(–CO–NH–).

**Ligand(II)**

The spectrum showed strong bands at 1717 and 1581 cm^−1^ in the spectrum of the Schiff base assigned to *υ*(C=O) of carbonyl and azomethine *υ*(C=N) vibrations, respectively. A broad medium intense bands were at 2959 and 2873 cm^−1^ due to methyl groups and band at 1136 cm^−1^ is assigned to hydrazinic *υ*(N–N) of the Schiff base [21–25].

**Figure 2:**
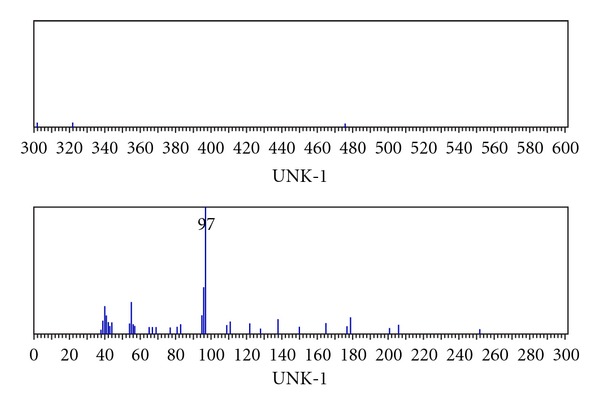
Mass spectra of Ligand(II).

**Figure 3:**
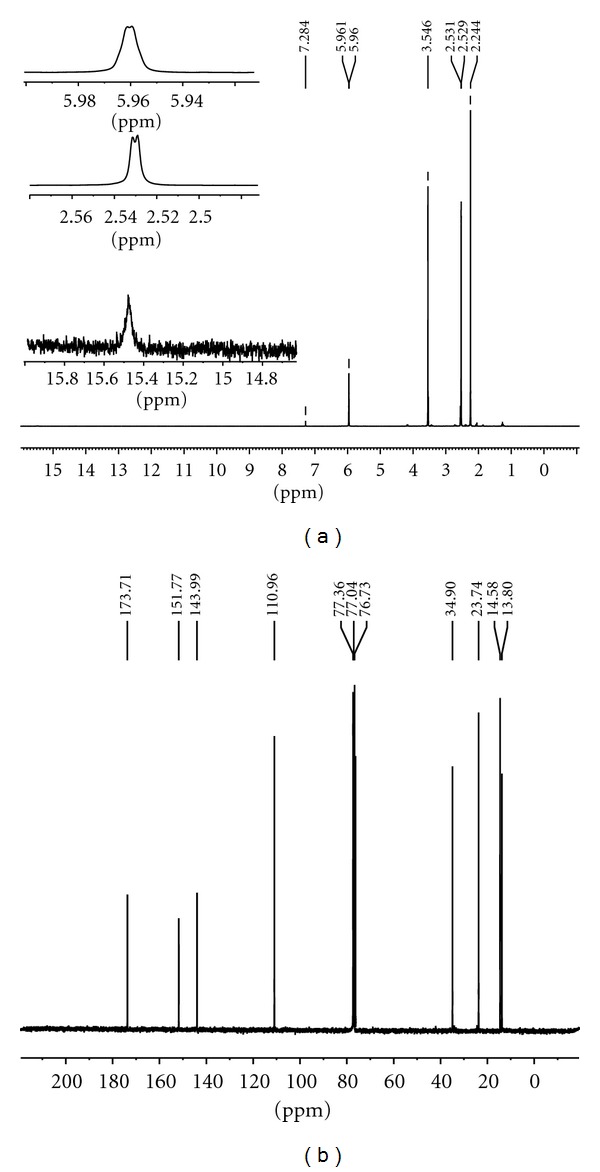
(a) ^1^H NMR spectra and (b) ^13^C-NMR spectra of Ligand(I).

In the electron impact spectrum (Figure 2) of the ligand(II), we confirm the probable formula by showing a peak at 476 amu, corresponding to macrocyclic moiety [(C_22_H_36_N_8_O_4_), calculated atomic mass 476]. The series of peaks in the range, that is, 38, 39, 40, 41, 42, 43, 54, 55, 56, 96, 97, 111, 121, 138, 150, 165, 179, 201, 205, 251, 301, 302 amu, and so forth, may be assigned to various fragments. Their intensity gives an idea of stability of fragments (Figure 2).

**Figure 4:**
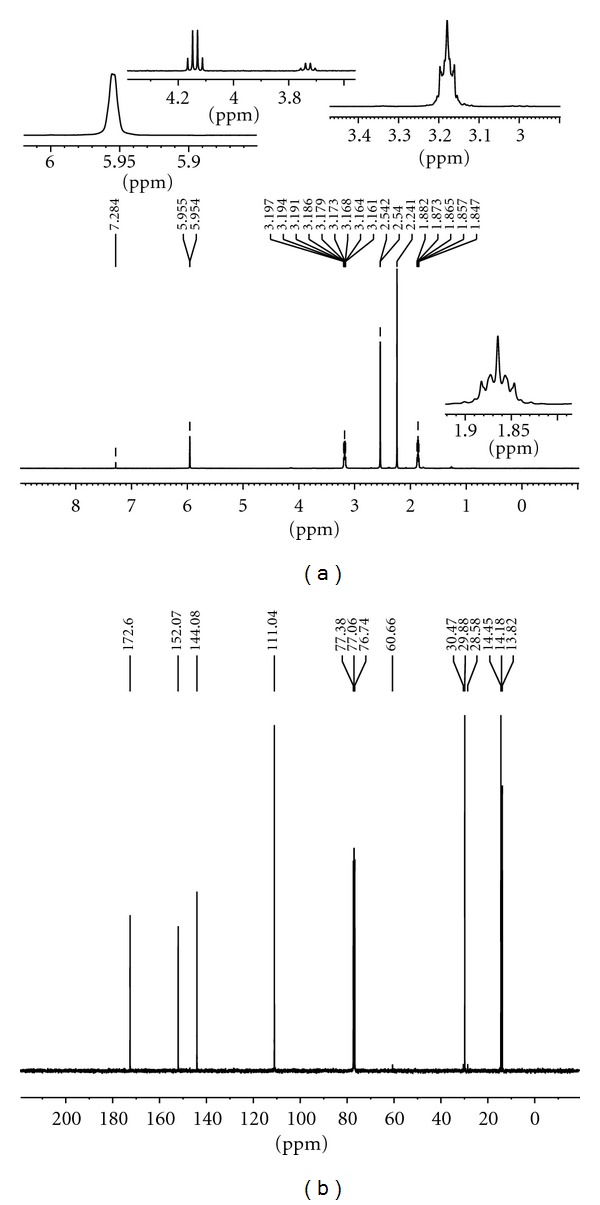
(a) ^1^H NMR spectra and (b) ^13^C-NMR spectra of Ligand(II).

In the ^1^H NMR spectrum, ligand(II) exhibits signals at 2.24 due to CH_3_–C(12H), 3.17 ppm due to –CO–CH_2_(4H), 1.8 ppm due to –CO–CH_2_–CH_2_(8H), 2.54 ppm due to –CH_2_–CN–(4H), and 5.9 ppm due to NH protons signal (Figure 4(a)). All these observations support the infrared conclusions.

In the ^13^C NMR spectrum Figure 4(b) of ligand, indicated new resonances are 13.82, 14.18, 14.45, 28.58, 29.88, 30.47, 60.66(CH_2_), 111.04, 144.08(CH_2_CO), 152.07(N=C), and 172.60(–CO–NH–).

### 3.1. Extraction of Metal Ions with Schiff Bases

#### 3.1.1. Effect of pH and Solvents on the Extraction of Cu(II) and Cr(III)

To the best of our knowledge, the stability of a transition metal complex with a polydentate chelate ligand in organic phase depends on a range of factors including: number and type of the donor atoms present, the number and size of the chelate rings formed on complexation [24]. In addition, the stability and selectivity of complexations strongly depend on the donor ability, dielectric constant of the solvent, and shape and size of the solvent molecules [26].

In this research, Liquid-liquid extraction experiments were performed to examine the efficiency and selectivity of hydrazones (I) and (II) in transferring s-metal ions (Li^+^, Na^+^, and K^+^) and d-metal ions (Cu^2+^ and Cr^3+^) from aqueous phase into chloroform. The results show that the alkali metals ions are not extracted by hydrazones (*E* < 3.5%).

**Figure 5:**
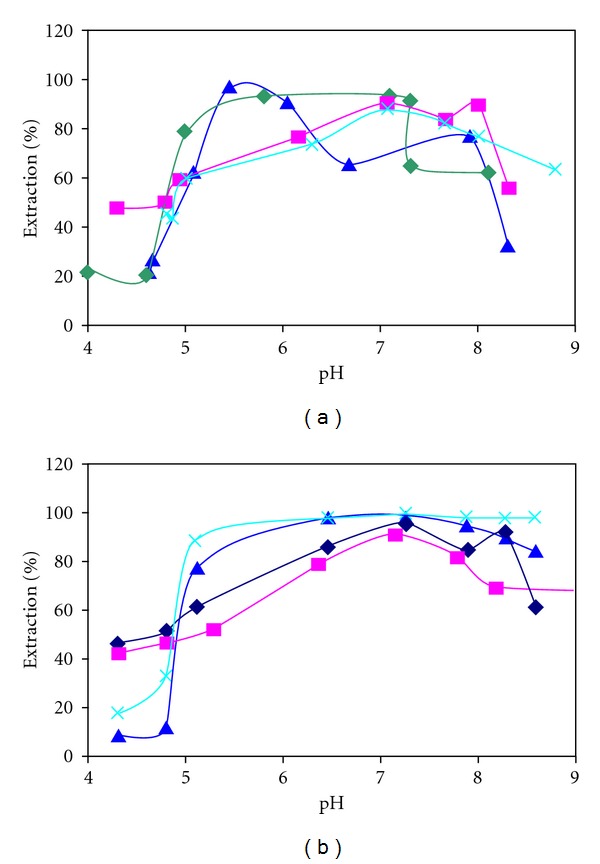
(a) Effect of pH on the extraction of Cu(II) and Cr(III). [Metal ions] = 1.5 × 10^−3^ M, [Ligand(I)] = 4 × 10^−4^ M in chloroform and dichloromethane. (▲) Chloroform(Cu) (*◆*) dichloromethane(Cu) (■) dichloromethane (Cr) (×) Chloroform (Cr). (b) Effect of pH on the extraction of Cu(II) and Cr(III). [Metal ions] = 1.5 × 10^−3^ M, [Extractant(II)] = 4 × 10^−4^ M in chloroform and dichloromethane. (▲) Chloroform(Cu) (×) dichloromethane(Cu) (■) Chloroform(Cr) (*◆*) dichloromethane(Cr).

 Also the results (Figures 5(a) and 5(b)) show the extractability of Cu^2+^ and Cr^3+^ from the aqueous phase into organic phase by macrocyclic hydrazones (I) and (II). It is clear that the extractability results of hydrazones are different for both of the organic solvents. When dichloromethane was used as organic solvent, macrocyclic Schiff bases extracted Cu^2+^ ion 98% and Cr^3+^ 94%. It is interesting that the percentage of the extraction of the metal ions with both hydrazones (I) and (II) is high. These results suggest that N donor (the presence of the lone electron pair on the nitrogen atom in hydrazone molecules provides their basic properties) increases the percentage of the extraction of the metal ions.

It can be seen from (Figures 5(a) and 5(b)) that the solvent has an important effect upon the cation extractability. The dielectric constants of dichlor.

omethane and chloroform are 9.1 and 4.8, respectively. Dichloromethane having a high dielectric constants is favored for the extraction of all the metal ions; there are similar results in the literature [27]. On the other hand, the better solvation of the complexes by dichloromethane may be a valuable reason for better extraction.

From the extraction data shown in the figures, it is clear that the ligands which have N_8_O_4_ donor sets show that both of the cationcavity size and the type of binding sites in the ring contribute to the ability of Cu^2+^ ions and Cr^3+^ ions binding. As shown in the figures, the copper and chrome extraction is high within the pH range of 5.2–6, 5–7(Cu), 7-8, 7–7.5(Cr), respectively. Moreover, transition metals were extracted in the order Cu > Cr, which are in the same order of decreasing ionic radius. 

#### 3.1.2. Composition of the Extracted Species

If only mononuclear species are extracted, under the condition in which chloride does not take part in the distribution equilibrium, the extraction process may be represented by equation

(2) $${{\text{Cu}}^{2+}}_{\left(w\right)}+{\text{H}}_{n}{\text{L}}_{\left(o\right)}⇌{\text{CuL}}_{\left(o\right)}+{{\text{nH}}^{+}}_{\left(w\right)},$$

where H_*n*_L (I) represents the extractant reagent and subscripts (*w*) and (*o*) denote the aqueous and organic phases, respectively. The extraction constant of the species CuL is given by

(3) K  ext  =[CuL]o[H+]wn[Cu2+]w[HnL]o.

When CuL is the only extractable species and the metal is present in the aqueous phase predominantly as the cation Cu^2+^, the metal distribution ratio (*D*) and the extraction constant are related by

(4) $$\log D=\log k_{\text{ext}}+n\text{pH}+\log{\left[{\text{H}}_{2}\text{L}\right]}_{o}.$$

**Figure 6:**
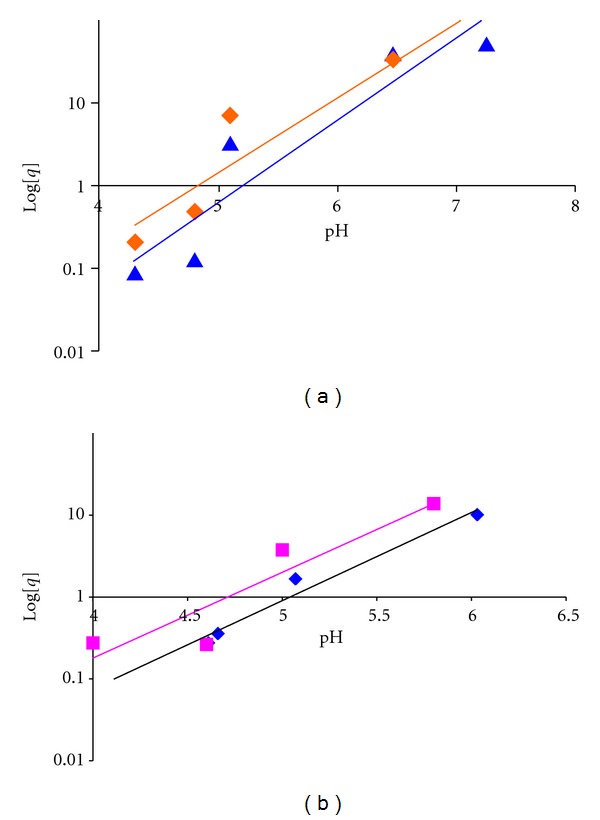
(a) The plot of log⁡*D* versus pH at constant Ligand(I). (■) chloroform (*◆*) dichloromethane. (b) The plot of log⁡*D* versus pH at constant Ligand(II). (*◆*) chloroform (▲) dichloromethane.

The effect of pH on the extraction of Cu^2+^ and Cr^3+^ ions from KCl media of ionic strength (*I* = 0.1 M) has been studied, the logarithm of the *D* values obtained was plotted against the corresponding pH values (according to (4) a plot of log⁡*D* against pH at constant 4 × 10^−4^ M of [H_*n*_L]). A straight line with a slope of about 2 was obtained at *I* = 0.1 of Cu^2+^ and Cr^3^, as shown Figures 6(a) and 6(b). The values represent the number of hydrogen ions released during the formation of metal-ligand complex and intercept log⁡[H_*n*_L] + log⁡*K*_ext_.

**Figure 7:**
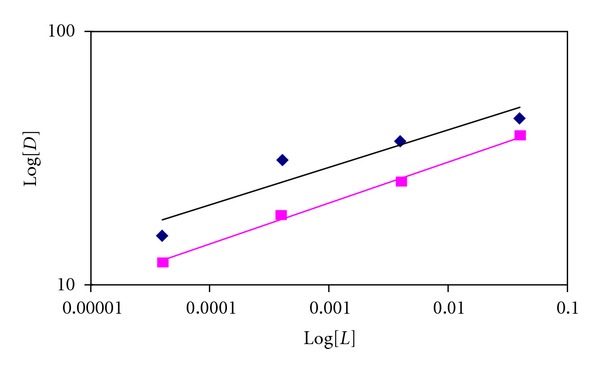
Log[*D*] versus log⁡[*L*] for the extraction of CuCl_2_ with ligand(I) (■) and Ligand(II) (■). In chloroform.

Also Figure 7 shows the evolution of log⁡*D* when increasing the concentration of ligands at constant chloride metal concentration with two different organic solvents. As seen from the plots, there is a linear relationship between log⁡*D* and log[L]org, and the slope should be equal to the number of ligand molecules per cation in the extracted species. The slopes of lines are equal to 0.5 of dichloromethane and chloroform. Therefore, ligands form a 1 : 2 (*L* : *M*) complex with Cu^2+^ for both solvents.

#### 3.1.3. Effect of Ionic Strength of Aqueous Phase

The influence of KCl in the concentration range of 0.1–1.0 M on the extraction efficiency of Cu^2+^ was studied in solutions containing 1.5 × 10^−3^ M Cu^2+^ and Cr^3+^ with 4 × 10^−4 ^M ligand in organic phase. The extraction efficiency decreases with increase in ionic strength of the aqueous medium. Taking into account (5), the extraction constant (*K*_ext_^0^) at zero ionic strength for this reaction can be correlated with the ionic strength(I) by

(5) $$\begin{array}{c} {{\text{Cu}}^{2+}}_{\left(w\right)}+{\text{H}}_{2}{\text{L}}_{\left(o\right)}⇌{\text{CuL}}_{\left(o\right)}+{{2\text{H}}^{+}}_{\left(w\right)}, \end{array}$$

(6) $$\begin{array}{c} K_{\text{ext}}^{0}=K_{\text{ext}}\frac{\gamma_{{\text{H}}^{+}}^{n}}{\gamma_{\text{C}{\text{u}}^{2+}}}, \end{array}$$

(7) $$\begin{array}{c} K_{\text{ext}}=K_{\text{ext}}^{0}\frac{\gamma_{\text{C}{\text{u}}^{2+}}}{\gamma_{{\text{H}}^{+}}^{n}}. \end{array}$$

According to the Debye-Huckel limiting law given

(8) $$\begin{array}{c} \log\gamma_{\pm }=-0.5Z_{i}^{2}\;\sqrt[{}]{I}. \end{array}$$

The activity coefficient (*γ*_±_) decreases with increase in ionic strength. At the constant pH, the activity coefficient of Cu^2+^ decreases as the ionic strength increase, hence *K*_ext_ decreases.

## 4. Conclusion

The new synthesized compounds act as tetra dentate Schiff bases. In most cases, these symmetrical compounds were obtained with yield more than 50% in some cases. Two imines (Schiff bases) were synthesized. We think that these compounds were prepared for the first time. This is confirmed by a precise review of the scientific background concerning this category of compounds. Their structures were identified by spectroscopy methods. The results indicate that H_2_L(I, II) in organic phase extracts efficiently Cu^2+^ and Cr^3+^ in aqueous phase containing 0.1 mol L^−1^ KCl in the pH range of approximately 5–7 and 7-8, respectively, at 25°C and 2 h stirring.
